# The Regulators Associated With N6-Methyladenosine in Lung Adenocarcinoma and Lung Squamous Cell Carcinoma Reveal New Clinical and Prognostic Markers

**DOI:** 10.3389/fcell.2021.741521

**Published:** 2021-12-09

**Authors:** Shuzhen Tan, Zesong Li, Kai Li, Yingqi Li, Guosheng Liang, Zhenye Tang, Jianhao Kang, Wenqing Chen, Minhua Li, Zhilin Zou, Guoliang Pi, Xiao Zhu

**Affiliations:** ^1^School of Laboratory Medicine, Hangzhou Medical College, Hangzhou, China; ^2^Southern Marine Science and Engineering Guangdong Laboratory (Zhanjiang), Guangdong Medical University, Zhanjiang, China; ^3^Guangdong Provincial Key Laboratory of Systems Biology and Synthetic Biology for Urogenital Tumors, Department of Urology, The First Affiliated Hospital of Shenzhen University, Shenzhen Second People’s Hospital (Shenzhen Institute of Translational Medicine), Shenzhen, China; ^4^Department of Radiation Oncology, Hubei Cancer Hospital, Tongji Medical College, Huazhong University of Science and Technology, Wuhan, China; ^5^Anhui Clinical and Preclinical Key Laboratory of Respiratory Disease, The First Affiliated Hospital of Bengbu Medical College, Bengbu, China

**Keywords:** lung cancer, N6-methyladenosine, prognosis, regulators, risk scores

## Abstract

N6-methyladenosine (m^6^A) methylation is of significant importance in the initiation and progression of tumors, but how specific genes take effect in different lung cancers still needs to be explored. The aim of this study is to analyze the correlation between the m^6^A RNA methylation regulators and the occurrence and development of lung cancer. The data of lung adenocarcinoma (LUAD) and lung squamous cell carcinoma (LUSC) were obtained through the TCGA database. We systematically analyzed the related pathological characteristics and prognostic factors by applying univariate and multivariate Cox regression, as well as LASSO Cox regression. Some of 23 m^6^A regulators are identified as having high expression in lung cancer. In addition, risk score has been shown to be an independent prognostic factor in lung cancer. Our research not only fully reveals that m^6^A regulators and clinical pathological characteristics are potentially useful with respect to survival and prognosis in different lung tumors but also can lay a theoretical root for the treatment for lung cancer—notably, to point out a new direction for the development of treatment.

## Introduction

M^6^A methylation, as a dynamic reversible process, is regulated by three enzymes: demethylase (erasers), function manager (readers), and methyltransferase complex (writers) (Yang et al., 2018; Zhou et al., 2020). Erasers include FTO and ALKBH5 (Li et al., 2017); readers include YTHDC1, YTHDC2, YTHDF1, YTHDF2, and HNRNPC (Zhang et al., 2019; He et al., 2020); and writers include MTETL3, METTL14, WTAP, KIAA1429, RBM15, and ZC3H13 (Tuncel and Kalkan, 2019; Asada et al., 2020). It is these m^6^A methylation regulators that are closely correlated with different human diseases, especially with cancer (Wang et al., 2018). A growing majority of evidence indicates that m^6^A has the capacity to exert a double effect in tumors. On the one hand, m^6^A regulates the expression of oncogenes and tumor suppressor genes, which act as promoting and suppressing cancer, respectively. On the other hand, the expression level of m^6^A and the expression and activity of m^6^A enzyme can be regulated to affect the function of m^6^A. Ultimately, they achieve the purpose of adjusting the differentiation of cancer stem cells and regulating T cell differentiation and immune homeostasis.

In the LUAD, the increased expression of METTL3 can enhance the translation of oncogene BRD4 by forming an mRNA ring with EIF3. With that, METTL3 accelerates the translation of oncogene by promoting ribosome recycling, thereby providing an easier way for the invasion and metastasis of lung cancer cells (Choe et al., 2018; Ni et al., 2021). The expression of FTO is elevated in the LUSC, and FTO inhibits cancer formation by inhibiting m^6^A methylation, which inhibits expression of the oncogene MZF1 eventually. Both m^6^A with high expression and MZF1 with deficient expression inhibit tumor development (Liu et al., 2018).

Generally speaking, patients with early treatment of lung cancer have a good prognosis, but most patients are diagnosed to be in the advanced stage of lung cancer, whose prognosis is poor (Wozniak, 2012; Zhu et al., 2019). Nowadays, TNM staging is still the dominant method to analyze the prognosis of lung cancer, although there is a certain degree of error and lack of individuality (Wozniak and Schwartz, 2018). Our preoccupation is searching for further accurate prognostic factors from the point of view of gene and molecule, for the sake of making accurate prognostic analysis and the individualized treatment selection.

In this article, we acquired the information of patients with lung adenocarcinoma (LUAD) and lung squamous cell carcinoma (LUSC), respectively. Pan-cancer data was the total of LUAD and LUSC data. The expression of m^6^A methylation regulators was analyzed to shed light on if it was related to LUAD, LUSC, and pan-cancer. Especially significant, based on these data, we try to find out the clinical correlation between m^6^A methylation regulators and lung cancer (Li et al., 2021).

## Results

### The Overview of m^6^A RNA Methylation Regulators in Lung Cancer

**FIGURE 1:**
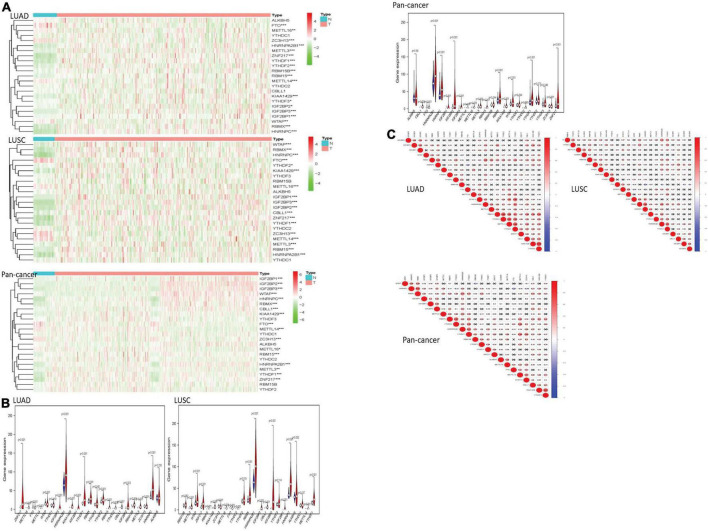
The overview of m^6^A RNA methylation regulators in lung cancer. **(A)** The figures reflect the expression levels of 23 m^6^A RNA methylation regulators in normal tissues and lung cancer tissues. When the gene expression level is high, the color turns red in the picture. However, the color becomes green with the gene expression decreasing. **(B)** The tree diagram at the top of the figure represents the clustering results of different samples from different experimental groups, and the tree diagram on the left demonstrates the clustering results of different genes from different samples. The “vioplots” clearly show the difference between m^6^A RNA methylation regulators in lung cancer and normal lung tissue. Blue represents normal lung tissue, and red represents lung cancer. **(C)** The diagram visually shows the relationship between different m^6^A RNA methylation regulators through Pearson correlation analysis. Red represents a positive correlation, and blue represents a negative correlation. *P < 0.05, **P < 0.01 and ***P < 0.01.

We drew a heatmap of LUAD, LUSC, and pan-cancer data after diff analysis firstly. Various gene expression levels were displayed as follows (Figure 1).

Most of the m^6^A RNA methylation regulators showed a weak to moderate positive correlation. KIAA1429 and YTHDF3 were the most correlated in LUAD. In LUSC, METTL3 and RBMX had the most obvious positive correlation. The negative correlation between HNRNPC gene and FTO gene was the most recognizable. In pan-cancer, KIAA1429 and YTHDF3 were the most relevant. HNRNPC and FTO were the most obvious negative correlation.

### Consensus Clustering of m^6^A RNA Methylation Regulators Identified Two Clusters of Lung Cancer

**FIGURE 2:**
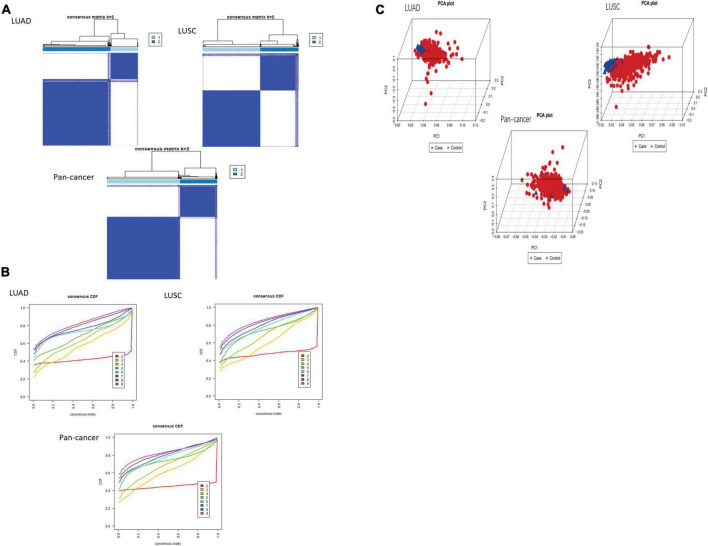
Classification of cluster by m^6^A RNA methylation regulator. **(A,B)** As K changed from 2 to 9, clustering cumulative distribution function of different lung cancer groups has been shown discriminatively. The consistent clustering matrix is the most suitable when *K* = 2. **(C)** PCR analysis results of the principal components are shown in the RNA expression profile among the TCGA database. Red represents lung cancer cases in cluster 1. Blue represents normal lung tissue of cluster 2.

After using “ConsensusClusterPlus” package to group LUAD, LUSC, and pan-cancer, the CDF value was small and the lines were smooth when *k* = 2. Next, PCA was used to verify whether the classification was correct. It was proved that the classification of cluster 1 and cluster 2 meets the requirements (Figures 2A–C).

### The Categories Determined by Consensus Clustering Are Closely Related to Clinical Results

**FIGURE 3:**
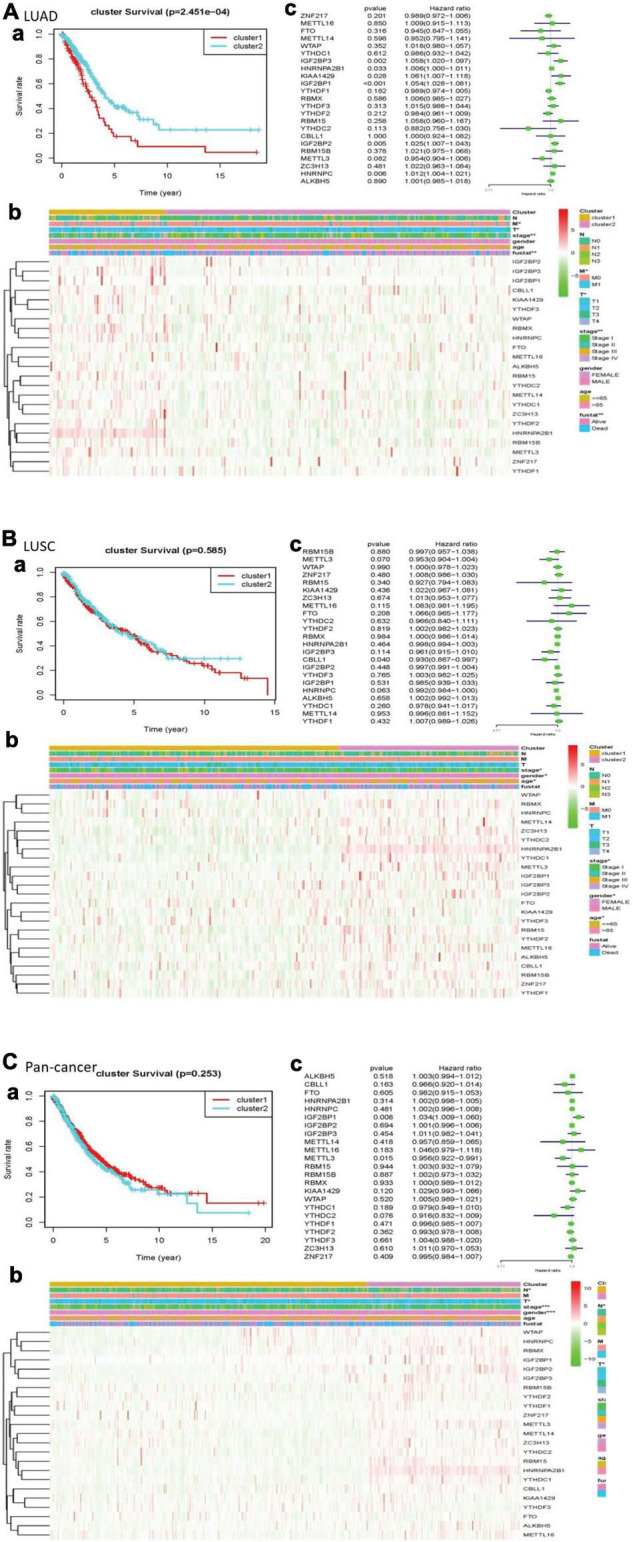
Clinical pathological characteristics and overall survival rate of cluster 1 and cluster 2 in different lung cancers, as well as the risk signature with m^6^A RNA methylation regulators. **(A)** LUAD; **(B)** LUSC; **(C)** pan-cancer. (a) Survival plot. The graph plots the overall survival curves of cluster 1 and cluster 2 in different lung cancers. Red represents cluster 1, and blue represents cluster 2. (b) Heatmap. The heat map shows the expression of m^6^A RNA methylation regulator in two clusters and its clinical pathological characteristics. When *P* < 0.05, the result is meaningful. (c) Univariate Cox regression. The graph records the hazard ratio and 95% confidence interval calculated by univariate Cox regression. The univariate analysis is beneficial to select the meaningful genes.

In the cleaned LUAD data, the survival probability of cluster 2 was greater than that of cluster 1. In assessing the relationship between clustering and clinical pathological features, the number of cluster 1 was significantly less than that of cluster 2. The result revealed that cluster 1 and cluster 2 had significant differences in M stage, T stage, total stage, and survival status. In LUSCs after washing, the difference in overall survival between the two clusters was not apparent. In the heatmap, the length of cluster 1 was slightly longer than that of cluster 2. There were differences in total stage, gender, and age between the two clusters. Of the cleaned pan-cancer, cluster 1 had a higher survival probability than cluster 2 when the patients had the same survival time. In the heatmap, the length of cluster 1 was also slightly longer than that of cluster 2, and there were differences in N stage, T stage, total stage, and gender (Figure 3).

### Prognostic Values of Risk Signature and m^6^A RNA Methylation Regulators

We used univariate Cox regression to get that, with these dangerous genes named IGF2BP3, HNRNPA2B1, KIAA1429, IGF2BP1, IGF2BP2, and HNRNPC, patients hardly survived. In LUSC, CBLL1 is a risk gene. In pan-cancer, IGF2BP1 was a risk gene, while METTL3 was a protective gene (Figure 3).

**FIGURE 4:**
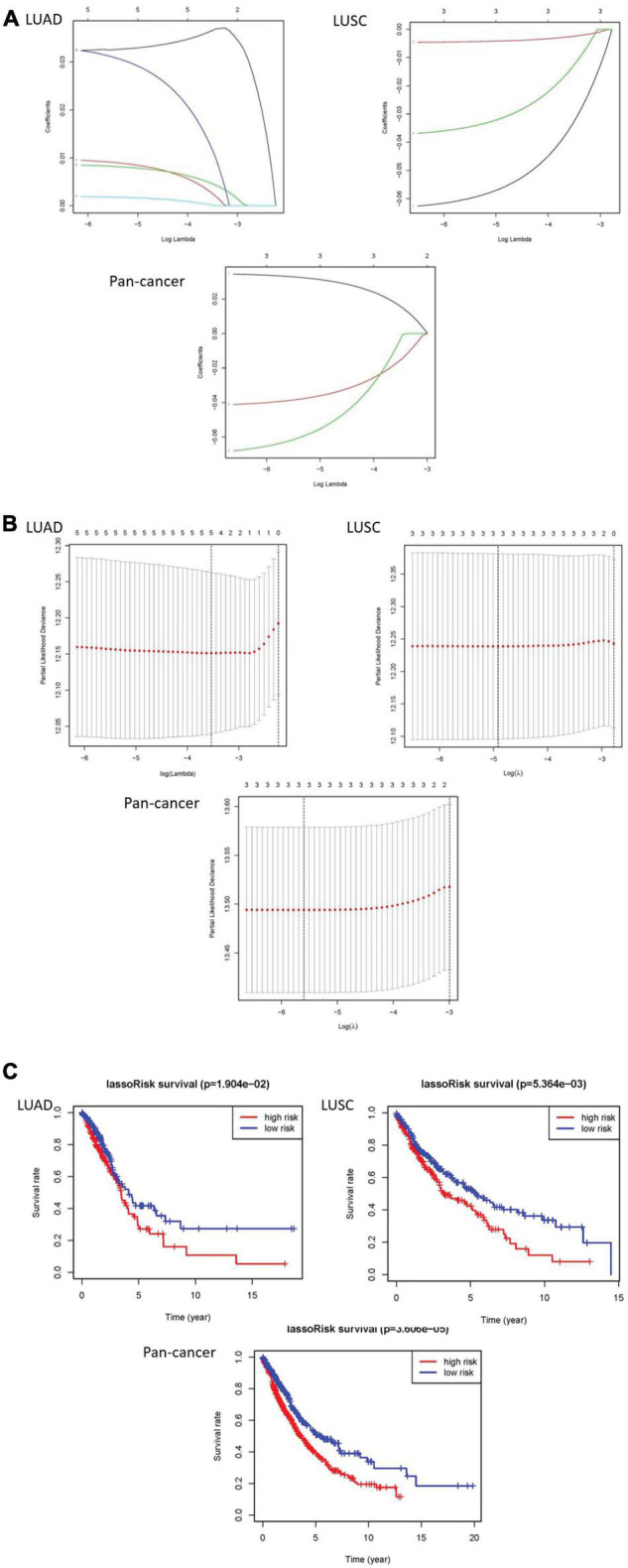
The results of LASSO Cox regression algorithm and survival curves. **(A,B)** Show coefficient results derived from LASSO Cox regression algorithm. **(C)** There are distinct differences of overall survival curves in the high-risk group and low-risk group which are distributed by risk score.

The least absolute shrinkage and selection operator (LASSO) Cox regression algorithm was implemented to analyze 23 genes. According to the minimum standard, 5 genes were selected for LUAD, 3 for LUSC, and 3 for pan-cancer. Five genes in LUAD are IGF2BP1, IGF2BP2, HNRNPC, KIAA1429, and HNRNPA2B1. Three genes are CBLL1, HNRNPC, and METTL3 in LUSC. In pan-cancer, three genes are IGF2BP1, METTL3, and YTHDC2. Then, risk characteristics were established, respectively. The coefficients were used to calculate the risk scores of each group. Among LUAD, LUSC, and pan-cancer, the survival rate of patients in the high-risk group is both lower than that in the low-risk group (Figures 4A–C).

### Prognostic Risk Scores Are Related to the Characteristics of Lung Cancer Clinical Cases

**FIGURE 5:**
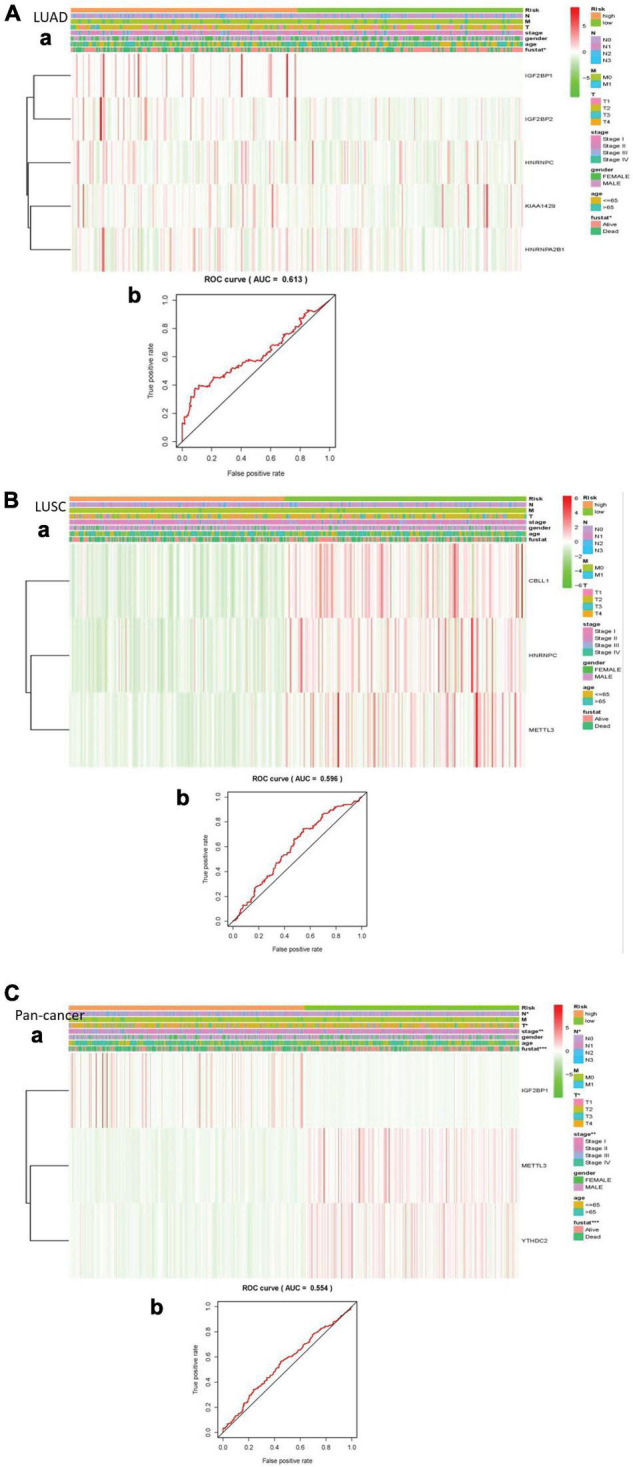
Explain the relationship between risk score, m^6^A RNA methylation regulator expression level, clinical pathological characteristics, risk group, and overall survival rate. **(A)** LUAD; **(B)** LUSC; **(C)** pan-cancer. (a) Every ROC curve indicates the relationship between risk score and survival rate, reflecting the predictive efficiency of prognostic signatures. (b) Each heatmap shows the expression level of m^6^A RNA methylation regulator in lung cancer of two clusters. In addition, there are apparent differences in the distribution of clinical pathological characteristics between the two groups. When *P* < 0.001, the result is significant.

We made a ROC curve to get the results that the risk score can be served as an independent prognostic factor. Next, a heatmap showed that there is a significant difference in survival status in LUAD. Similarly, the pan-cancer data have obvious differences in N stage, T stage, total stage, and survival status. However, there are no significant differences in any pathological features of LUSC (Figures 5A–C).

**FIGURE 6:**
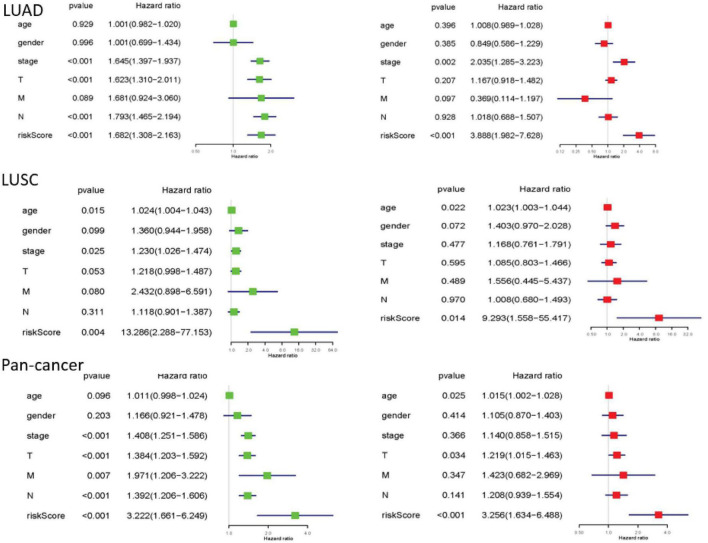
The results of univariate and multivariate Cox regression analysis. Univariate and multivariate Cox regression analysis between overall survival rate and clinical pathological characteristics was performed for each type of lung cancer. They could identify the independent prognostic factors. When *P* < 0.05, the result is meaningful.

Finally, we performed univariate and multivariate Cox regression analysis. In univariate analysis, total stage, T stage, N stage, and risk scores are all related to overall survival in LUAD. Age, total stage, and risk scores are connected with overall survival in LUSC. Total staging, T staging, M staging, N staging, and risk scores have something to do with overall survival in pan-cancer. When all factors were incorporated into the multivariate analysis, the total stage and risk scores were still significantly correlated with the overall survival rate in LUAD. Age and risk scores are related to overall survival in LUSC. T staging and risk scores were in connection with overall survival in pan-cancer (Figure 6).

## Discussion

With the increasing incidence and mortality of lung cancer today, there are quite in-depth discoveries in DNA and protein modification, so the treatment of lung cancer is also updated at an alarming rate (Karlsson et al., 2019; Siegel et al., 2020). This study explored the clinical prognostic effect of m^6^A in LUAD, LUSC, and pan-cancer for the purpose of laying the foundation for treatment strategies based on RNA methylation.

In this study, we found that most of the 23 m^6^A RNA methylation regulators were abnormally expressed in LUAD, LUSC, and pan-cancer. Among them, KIAA1429 and YTHDF3 showed a significant positive correlation. YTHDF3 is a direct m^6^A reader, which can promote the translation of target mRNAs and improve their stability. The proliferation, migration, and invasion of cancer cells were enhanced with the upregulated expression of METTL3 (Cheng et al., 2019). When the expression level of METTL3 is upregulated, it enhances the proliferation, migration, and invasion of cancer cells *in vitro* and the growth of tumors *in vivo*. Meanwhile, YTHDF3 collaborates with YTHDF1 to promote protein synthesis and influence methylated mRNA decay mediated by YTHDF2. Together with YTHDF1 and YTHDF2, YTHDF3 plays a key role in accelerating metabolism of mRNAs modified by m^6^A in the cytoplasm (Wang X. et al., 2014; Li et al., 2017; Shi et al., 2017).

In the LUAD, LUSC, and pan-cancer, there are significant differences in the overall survival of the two different clusters. They also have significant correlation in different clinical pathological features, indicating that the level of m^6^A RNA methylation regulator is distinctly related to the poor prognosis of lung cancer.

In the LUAD, KIAA1429 is associated with the formation of m^6^A methyltransferase complex (MTC), which promotes the formation of m^6^A (Bokar et al., 1997; Ping et al., 2014; Wang Y. et al., 2014). HNRNPC is considered as the promoter of tumorigenesis, and overexpression of HNRNPC can be found in the main regulators of various cancer progression-related genes, which is closely connected to the poor clinical prognosis of cancer (Huang et al., 2016; Fischl et al., 2019). The risk gene for LUSC is CBLL1. In addition, pan-cancer’s risk gene is METTL3 and the protective gene is IGF2BP1. METTL3 can participate in the composition of MTC as well. Cytoplasmic METTL3 is served as a m^6^A reader to promote the translation of target mRNA (Liu et al., 2014; Cheng et al., 2019; Ru et al., 2020; Shi et al., 2020). This result is used as a basis for predicting the survival rate of lung cancer according to the genetic differences of patients in the future. Even the known gene variation could be used to fundamentally treat lung cancer from the genetic perspective, for example, some medicine will target m^6^A methylation (Deng et al., 2018).

We selected the appropriate gene according to the minimum standard and calculated their respective risk scores in the wake of dividing lung cancer into high-risk and low-risk categories by the LASSO algorithm. Then, the ROC curve and heatmap are used to verify that the risk scores can effectively predict the survival of lung cancer patients. Above all, risk scores provide an important basis for clinical evaluation of lung cancer prognosis and provide personalized treatment options.

In summary, this study systematically described the differences in the expression levels of m^6^A RNA methylation regulators between LUAD, LUSC, and pan-cancer data, revealing the prognostic role of their expressions in relation to the clinical pathological characteristics of lung cancer. M^6^A RNA methylation regulator obviously not only plays a vital role in the occurrence and development of lung cancer but also affects the clinical manifestations and prognostic development of lung cancer (Wang et al., 2021). Besides, risk scores can become a far-reaching factor in predicting the prognosis of lung cancer, providing essentially for clinical treatment guidance.

## Materials and Methods

### Data Acquisition

A total of 1037 cases of lung cancer were obtained from the TCGA database,^1^ including 535 cases of LUAD and 502 cases of LUSC. In addition, there were 108 cases in the control group, containing 59 cases of LUAD and 49 cases of LUSC.

### Selection of m^6^A RNA Methylation Regulators

Depending upon the literature (Shi et al., 2018; Li et al., 2019, 2020; Sun et al., 2019; Liu et al., 2020; Meng et al., 2020; Muthusamy, 2020; Zhou et al., 2020; Zhang et al., 2021; Zou et al., 2021), 23 tumor-related m^6^A RNA methylated modulators were selected. These 23 genes were extracted and systematically analyzed with the data of LUAD, LUSC, and pan-cancer in TCGA database. Through these procedures, we could search the relationship between the expression of 23 m^6^A-related genes and the clinical prognosis of lung cancer patients.

### Bioinformatic Analysis

In order to examine the role of m^6^A RNA methylation regulator in lung cancer, we first used “limma” package to analyze 23 m^6^A RNA methylation regulator related genes in each lung cancer group. In the next step, expression levels of 23 m^6^A RNA methylation regulators had been distinctly demonstrated by “diff” analysis, “pheatmap” package, and “vioplot” package. Correlation figures were drawn to illustrate the relationship between different m^6^A RNA methylation regulators. Besides, we utilized the “ConsensusClusterPlus” package to delete the normal lung tissue data in each lung cancer group and divide the remaining lung cancer data into two groups. PCA could verify whether the classification was correct. The survival software package had the capability to study the survival of grouped lung cancers and draw a clinical heatmap of the correlation between different clusters and the relationship between survival and clinical data.

More than anything, for the purpose of finding the relationship between the gene and survival and its effect on the prognosis, the m^6^A RNA methylation Regulator genes were conducted by univariate Cox regression analysis and LASSO Cox regression algorithm (Bovelstad et al., 2007; Sauerbrei et al., 2007).

**FIGURE 7:**
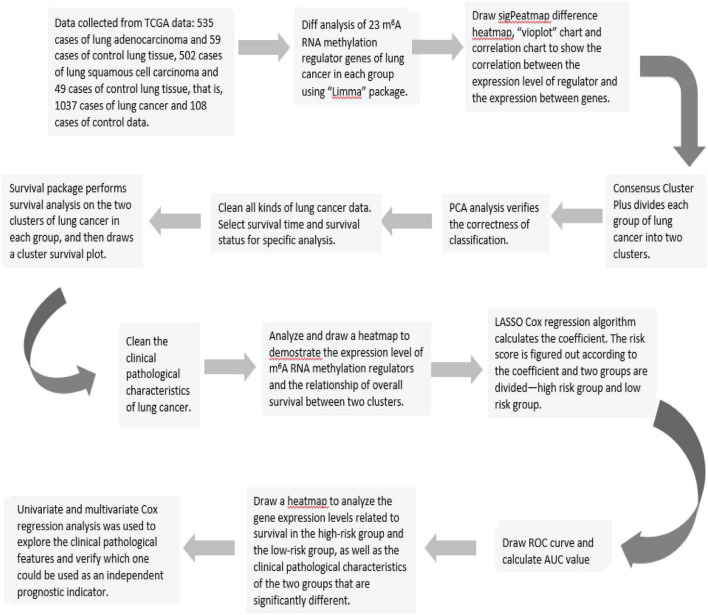
Flow chart for analyzing and processing lung cancer data in the TCGA data set.

In the end, we could construct the survival-related linear risk assessment model—risk scores. Calculation formula: Risk score = $\sum_{i=l}^{N}E\,x\,p_{l}\times w_{l}$. Exp in the formula represents the expression level of the gene, and w represents the coefficient of the gene in the univariate or multivariate Cox regression analysis. ROC curve and heatmap were assessed if risk score could be a prognostic factor in lung cancer (Figure 7).

### Statistical Analyses

“Diff” package was used to analyze the expression levels of 23 m^6^ARNA methylation modulator related genes in LUAD, LUSC, and pan-cancer. Then, the “ConsensusClusterPlus” software package was used to treat LUSC, LUSC, and total lung cancer. Also, each group of lung cancer was divided into two clusters. With the help of univariate Cox regression analysis, the relationship between different clusters and clinical data was clarified. At the same time, the prognostic effect of related genes was analyzed in combination with multivariate regression analysis. Finally, LASSO Cox regression algorithm was performed to analyze related genes and divide each group of lung cancer into high-risk group and low-risk group to find independent prognostic factors. Overall survival referred to the time from the date of diagnosis to death for any reason. This study used Practical Extraction and Report Language (Perl) and R version 3.5.3 for statistical analysis. The results were statistically significant when *P* < 0.05.

## Data Availability Statement

The original contributions presented in the study are included in the article/supplementary material, further inquiries can be directed to the corresponding author/s.

## Ethics Statement

The studies involving human participants were reviewed and approved by Guangdong Medical University Ethics committee. The ethics committee waived the requirement of written informed consent for participation.

## Author Contributions

XZ and GP: conceptualization, methodology, supervision, funding, and software. ST: data curation and writing—original draft preparation. ST, KL, GL, ZT, ZZ, and XZ: visualization and investigation. ST, ZL, YL, JK, WC, ML, and XZ: writing—reviewing and editing. XZ: checked the statistical accuracy as an expert in statistics and bioinformatics. All authors read and approved the final version of the manuscript.

## Conflict of Interest

The authors declare that the research was conducted in the absence of any commercial or financial relationships that could be construed as a potential conflict of interest.

## Publisher’s Note

All claims expressed in this article are solely those of the authors and do not necessarily represent those of their affiliated organizations, or those of the publisher, the editors and the reviewers. Any product that may be evaluated in this article, or claim that may be made by its manufacturer, is not guaranteed or endorsed by the publisher.

## Footnotes

1
https://cancergenome.nih.gov/
