# Corrigendum: The Regulators Associated With N6-Methyladenosine in Lung Adenocarcinoma and Lung Squamous Cell Carcinoma Reveal New Clinical and Prognostic Markers

**DOI:** 10.3389/fcell.2022.843275

**Published:** 2022-03-07

**Authors:** Shuzhen Tan, Zesong Li, Kai Li, Yingqi Li, Guosheng Liang, Zhenye Tang, Jianhao Kang, Wenqing Chen, Minhua Li, Zhilin Zou, Guoliang Pi, Xiao Zhu

**Affiliations:** ^1^ School of Laboratory Medicine, Hangzhou Medical College, Hangzhou, China; ^2^ Southern Marine Science and Engineering Guangdong Laboratory (Zhanjiang), Guangdong Medical University, Zhanjiang, China; ^3^ Guangdong Provincial Key Laboratory of Systems Biology and Synthetic Biology for Urogenital Tumors, Department of Urology, The First Affiliated Hospital of Shenzhen University, Shenzhen Second People’s Hospital (Shenzhen Institute of Translational Medicine), Shenzhen, China; ^4^ Department of Radiation Oncology, Hubei Cancer Hospital, Tongji Medical College, Huazhong University of Science and Technology, Wuhan, China

**Keywords:** lung cancer, N6-methyladenosine, prognosis, regulators, risk scores

In the published article, there was an error in affiliation 1. Instead of “Anhui Clinical and Preclinical Key Laboratory of Respiratory Disease, Department of Respiratory Disease, The First Affiliated Hospital of Bengbu Medical College, Bengbu, China”, it should be “School of Laboratory Medicine, Hangzhou Medical College, Hangzhou, China”.

The authors apologize for this error and state that this does not change the scientific conclusions of the article in any way. The original article has been updated.

